# Fibroblasts from bank voles inhabiting Chernobyl have increased resistance against oxidative and DNA stresses

**DOI:** 10.1186/s12860-018-0169-9

**Published:** 2018-08-29

**Authors:** Venla Mustonen, Jenni Kesäniemi, Anton Lavrinienko, Eugene Tukalenko, Tapio Mappes, Phillip C. Watts, Jaana Jurvansuu

**Affiliations:** 1 Department of Ecology and Genetics, University of Oulu, FI-90014 Oulu, Finland; 2 Institute of Biology and Medicine, Taras Shevchenko National University of Kyiv, Kyiv, UA-03022 Ukraine; 3 Department of Biological and Environmental Science, University of Jyväskylä, FI-40014 Jyväskylä, Finland

**Keywords:** Bank vole, Chernobyl, Environmental ionizing radiation, p53, DNA damage, Antioxidant capacity

## Abstract

**Background:**

Elevated levels of environmental ionizing radiation can be a selective pressure for wildlife by producing reactive oxygen species and DNA damage. However, the underlying molecular mechanisms that are affected are not known.

**Results:**

We isolated skin fibroblasts from bank voles (*Myodes glareolus*) inhabiting the Chernobyl nuclear power plant accident site where background radiation levels are about 100 times greater than in uncontaminated areas. After a 10 Gy dose of gamma radiation fibroblasts from Chernobyl animals recovered faster than fibroblasts isolated from bank voles living in uncontaminated control area. The Chernobyl fibroblasts were able to sustain significantly higher doses of an oxidant and they had, on average, a higher total antioxidant capacity than the control fibroblasts. Furthermore, the Chernobyl fibroblasts were also significantly more resistant than the control fibroblasts to continuous exposure to three DNA damaging drugs. After drug treatment transcription of p53-target gene pro-apoptotic Bax was higher in the control than in the Chernobyl fibroblasts.

**Conclusion:**

Fibroblasts isolated from bank voles inhabiting Chernobyl nuclear power plant accident site show elevated antioxidant levels, lower sensitivity to apoptosis, and increased resistance against oxidative and DNA stresses. These cellular qualities may help bank voles inhabiting Chernobyl to cope with environmental radioactivity.

**Electronic supplementary material:**

The online version of this article (10.1186/s12860-018-0169-9) contains supplementary material, which is available to authorized users.

## Background

The Chernobyl nuclear power plant disaster in 1986 was classified by the International Atomic Energy Agency to the most severe radiation accident level. Since then, the most contaminated area around the nuclear power plant has been closed to the general public. This Chernobyl exclusion zone covers about 2600 km^2^ and still contains patches of radioactively contaminated soil emitting from normal background levels of 0.2 μSv/h up to about 200 μSv/h [1].

A meta-analysis has shown that ionizing radiation has increased mutation frequency in various Chernobyl taxa as represented by, for example, discolorations, cataracts, chromosomal abnormalities, or cancers [2]. Another meta-analysis on wildlife inhabiting Chernobyl area found a small to intermediate increase in oxidative damage (such as imbalance between oxidants and antioxidants) and a decrease in antioxidant defenses (such as depleted antioxidant levels) [3]. However, the prevalence of these effects varied among species, implying that there is no standard response to chronic low-dose radiation and that some species are more radioresistant than others. Accordingly, the species abundance in Chernobyl area has been reported to be in decline, constant, and on rise, due to either the adverse effects of ionizing radiation or because of lack of detrimental human activity [4–7]. Furthermore, evidence for an adaptive response to chronic low-dose radiation at Chernobyl is somewhat equivocal [8]. Nonetheless, adaptation has been reported in plants: plants within Chernobyl area have higher resistance to DNA-damaging chemicals, antioxidants, osmotic stress, and gamma irradiation than control plants and have also changes in antioxidant and stress responses, and in DNA repair [9–11].

The bank vole (*Myodes glareolus*) is an ideal mammalian model to study the effects of environmental radiation in Chernobyl, because it is common at the site, remains close to the surface source of radiation, and as a small animal has relatively small territory. Bank vole population sizes were very low following the accident at Chernobyl but numbers of this species rebounded during the following year, probably via immigration from several nearby populations [12, 13]. Since the accident, Chernobyl bank voles have been shown to harbor slight but significant increase in amounts of chromosomal aberrations, mitochondrial DNA mutations, and cataracts in females, yet other work has found no signs of genotoxic stress [14–17].

Ionizing radiation can cause long-lasting and inherited effects by damaging DNA directly or via water-derived free radicals. To determine whether exposure to chronic low-dose radiation extending possibly several generations has affected bank vole cells’ ability to tolerate oxidative and DNA stress we isolated skin fibroblasts from bank voles from the Chernobyl exclusion zone and from an uncontaminated control area near Kiev. We chose to study skin fibroblasts because they are easy to isolate even in field conditions. The fibroblasts were challenged with gamma radiation to study their reactions to the original type of stress, and with an oxidative agent and three DNA damaging drugs to dissect the responses to two biologically important aspects of ionizing radiation: oxidative stress and DNA damage. In all treatments, Chernobyl fibroblasts were, on average, able to sustain the insults better than the control fibroblasts. Moreover, Chernobyl fibroblasts had higher total antioxidant capacity than the control cells and were less sensitive to DNA damage induced lethality, both of which processes may explain their increased resistance against radiation.

## Results

### Chernobyl fibroblasts recover after 10 Gy irradiation faster than control fibroblasts

**Fig. 1:**
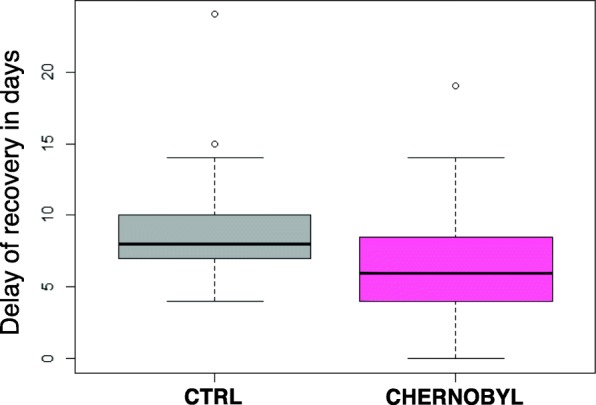
Chernobyl bank vole fibroblasts recover faster after irradiation than control cell lines. The fibroblast cell lines isolated from Chernobyl bank voles (pink) were able to reach 100% confluency, on average, 2 days faster than the control cell lines (grey) after 10 Gy irradiation. “The delay of recovery” represents how many days longer an irradiated cell line took to cover the plate than the corresponding non-irradiated cell line. The figure shows results from three separate experiments using eight Chernobyl (*N* = 24) and eight control (*N* = 24) cell lines. In the box blot the box presents the lower and upper quartiles, median is indicated by a line, whiskers show values within 1.5 interquartile range from the boxed values, and circles are outliers (R version 3.3.3)

Skin fibroblasts were isolated from eight male bank voles from the Chernobyl exclusion zone (an average dose rate of 21 μSv/h) and from an uncontaminated control area near Kiev (an average dose rate of 0.2 μSv/h). The same number of fibroblasts for each cell line were irradiated at the rate of 3.5 Gy/min from a cesium-137 source. The cells were allowed to recover for 24 h and then replated to a flask with about 4.5 times bigger growth surface area. After 24 h of the irradiation, we did not see increase in apoptotic or floating dead cells in any of the cell lines and thus we chose a growth based assay instead of a viability test to study the cells. A clonogenic survival assay, typical for these kind of experiments, was not suitable for these primary fibroblasts because they readily die if plated too scarcely. Recovery date was scored when cells reached 100% confluency. The delay of recovery (Fig. 1) was calculated by how many days longer the irradiated cell line took to reach confluency in comparison to the untreated cell line. Fibroblasts from Chernobyl animals were significantly (Student’s t-test *p*-value = 0.028) faster to recover after the irradiation than fibroblasts from control animals. Control fibroblasts took on average 9.2 days longer to fill the plate after 10 Gy irradiation whereas Chernobyl cells took just 6.8 days longer than the corresponding untreated cell lines.

### Chernobyl fibroblasts grow in higher concentrations of oxidant and have higher total antioxidant capacity than control fibroblasts

**Fig. 2:**
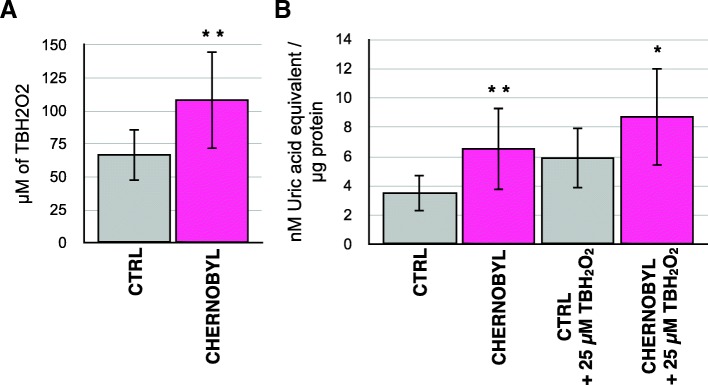
Chernobyl fibroblasts survive in higher oxidant concentration and have more antioxidants than control fibroblasts. **a** Fibroblasts isolated from Chernobyl bank voles were, on average, able to grow in about 40 μM higher concentration of oxidizing agent tert-butyl hydroperoxide (TBH2O2) than control bank vole fibroblasts. Cells were treated with different concentrations of the oxidant for 24 h after which fresh cell culture media was changed and cells were let grow for 7 days before scoring the wells that remained 100% confluent. **b**. Chernobyl fibroblasts have significantly higher total antioxidant capacity in untreated and oxidant-treated cells than control fibroblasts. For oxidant treatment cells were grown in 25 μM of TBH2O2 for 24 h before the total antioxidant capacity was measured. Results are from three separate experiments using eight Chernobyl (*N* = 24) and eight control (*N* = 24) cell lines, variation is shown by standard deviation, and statistical analysis was done with Student’s t-test (* = *p* ≤ 0.05, ** = *p* ≤ 0.01)

To determine whether Chernobyl fibroblasts are more resistant to the oxidative or the DNA damaging effect of radiation we first studied the effect of the oxidant tert-butyl hydroperoxide on the cells. Cells were plated on 24 well plates at 80% confluency and exposed to different concentrations (50–150 μM) of tert-butyl hydroperoxide for 24 h before changing the media. Cells were allowed to grow for a week and then 100% confluent wells were scored (Fig. 2a). Chernobyl fibroblasts were able to recover and grow in about 40 μM higher concentration of the oxidant than the control fibroblasts (x_CTRL_ = 66.7 μM, x_CHERNOBYL_ = 108.3 μM, Student’s t-test *p*-value = 0.0096). We also tested whether the cells would differ in response to constant exposure to small concentrations (10–50 μM) of the oxidant added every other day for four times but both groups, except one control cell line, were able to adjust and grew in all the concentrations (Additional file 1).

To assess if Chernobyl fibroblasts tolerate more oxidant because they had more antioxidants, total antioxidant levels were measured by cell extracts’ capacity to reduce copper. Cell extracts from cells grown in normal conditions or after 24 h challenge with 25 μM of tert-butyl hydroperoxide we tested (Fig. 2b). On average, Chernobyl fibroblasts had almost twice the total antioxidant capacity in normal cell culture conditions than the control cells (x_CTRL_ = 3.5 nM/μg, x_CHERNOBYL_ = 6.5 nM/μg, *p*-value = 0.010). Total antioxidant capacity in the oxidant-challenged cell lines was greater than in unchallenged cell lines in both groups and the increase was similar: 2.4 and 2.2 units for control and Chernobyl cells, respectively. The similar level of antioxidant induction in the two groups suggests that regulatory responses in Chernobyl fibroblasts were intact. However, Chernobyl fibroblasts had still significantly higher total antioxidant capacity (*p*-value = 0.041) than the control cells after the antioxidant treatment. The higher basal antioxidant levels in Chernobyl fibroblasts may protect the cells against excess oxidants generated by ionizing radiation exposure.

### Chernobyl fibroblasts grow in higher concentrations of DNA damaging drugs than control fibroblasts

**Fig. 3:**
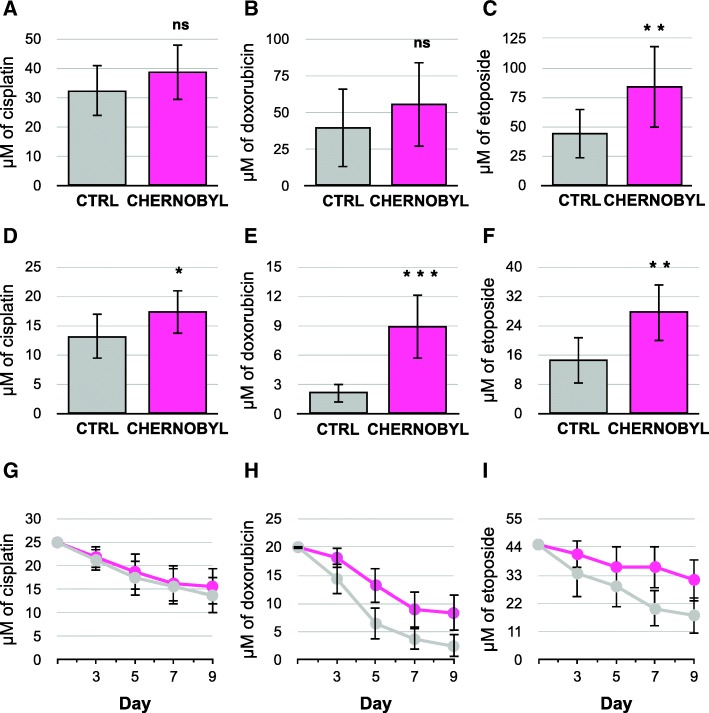
Chernobyl bank vole fibroblasts survive on higher concentrations of DNA damaging drugs than control cells. Cells were plated at 80% confluency and treated with different concentrations of the DNA damaging drugs for 24 h after which fresh cell culture media was changed and the cells were let to grow for 7 days before scoring the wells that were 100% confluent. Treatment with **a**.) cisplatin or **b**.) doxorubicin did not show significant difference between the Chernobyl and control cell lines, whereas Chernobyl cells were able to grow in, on average, about 30 μM higher concentrations of **c**.) etoposide. For constant exposure of DNA damaging drugs cells were treated with the drug every other day for four times before scoring the wells that were 100% confluent. Chernobyl fibroblasts were able to grow in significantly higher chronic concentrations of D.) cisplatin, **e**.) doxorubicin and **f**.) etoposide than control cells. Examples from one experiment (*N* = 8) on the effect of constant drug treatment on reduction of 100% confluent Chernobyl and control fibroblast cultures after treatment with **g**.) cisplatin, **h**.) doxorubicin, and **i**.) etoposide. Results, except **g**-**i**, are from three separate experiments using eight Chernobyl (*N* = 24) and eight control (*N* = 24) cell lines, variation is shown by standard deviation, and statistical analysis was done with Student’s t-test (ns = *p* > 0.05, * = *p* ≤ 0.05, ** = *p* ≤ 0.01, *** = *p* ≤ 0.001)

We tested the bank vole fibroblasts’ ability to withstand DNA damage by exposing cells to DNA cross-linking drug cisplatin, DNA-intercalating drug doxorubicin, and topoisomerase inhibitor etoposide. Cisplatin adducts are repaired mainly by nucleotide excision repair whereas doxorubicin and etoposide produce double-strand breaks that are subject to homologous recombination [18]. Cells were plated at 80% confluence and treated with different concentrations of the drugs for 24 h before replacing the media. The cells were allowed to grow for a week before scoring the concentration where they were able to grow to 100% confluency. Chernobyl fibroblasts tolerated significantly more of etoposide than the control cells (x_CTRL_ = 44.1 μM, x_CHERNOBYL_ = 83.6 μM, Student’s t-test *p*-value = 0.004; Fig. 3c). Yet there was no significant differences between the groups to the other drug treatments (Fig. 3a and b). Because a constant small amount of DNA damage is more appropriate condition to mimic the environmental radiation to which the Chernobyl bank voles are exposed, we treated the cells with low concentrations of the drugs every other day for four times and recorded the 100% confluent wells a day after the last treatment. Chernobyl cells were significantly more resistant to all DNA damaging drugs (Fig. 3d to f), especially doxorubicin and etoposide (Fig. 3e and f), than the control cells. On average, the Chernobyl fibroblasts were able to grow in over four times higher doxorubicin concentrations than the control cell lines (x_CTRL_ = 2.2 μM, x_CHERNOBYL_ = 9.0 μM, *p*-value < 0.0001). The difference in tolerance between Chernobyl and control cells treated with etoposide (x_CTRL_ = 14.6 μM, x_CHERNOBYL_ = 27.5. μM, *p*-value = 0.0015; Fig. 3f) and cisplatin (x_CTRL_ = 13.1 μM, x_CHERNOBYL_ = 17.2 μM, *p*-value = 0.018; Fig. 3e) were smaller but still significant. Chernobyl cells seemed more tolerant to dsDNA break producing drugs doxorubicin and etoposide than to cisplatin-derived adducts. In contrast to oxidant exposure, cells were incapable of adjusting to constant dose of DNA damaging drugs: the longer the cells were treated with the drugs, the more sensitive they became as shown by the chronic dose experiment where the 100% confluent wells were recorded a day after every treatment (Fig. 3g to i).

### After DNA damage Chernobyl fibroblasts remain arrested at G2 whereas control fibroblasts die

**Fig. 4:**
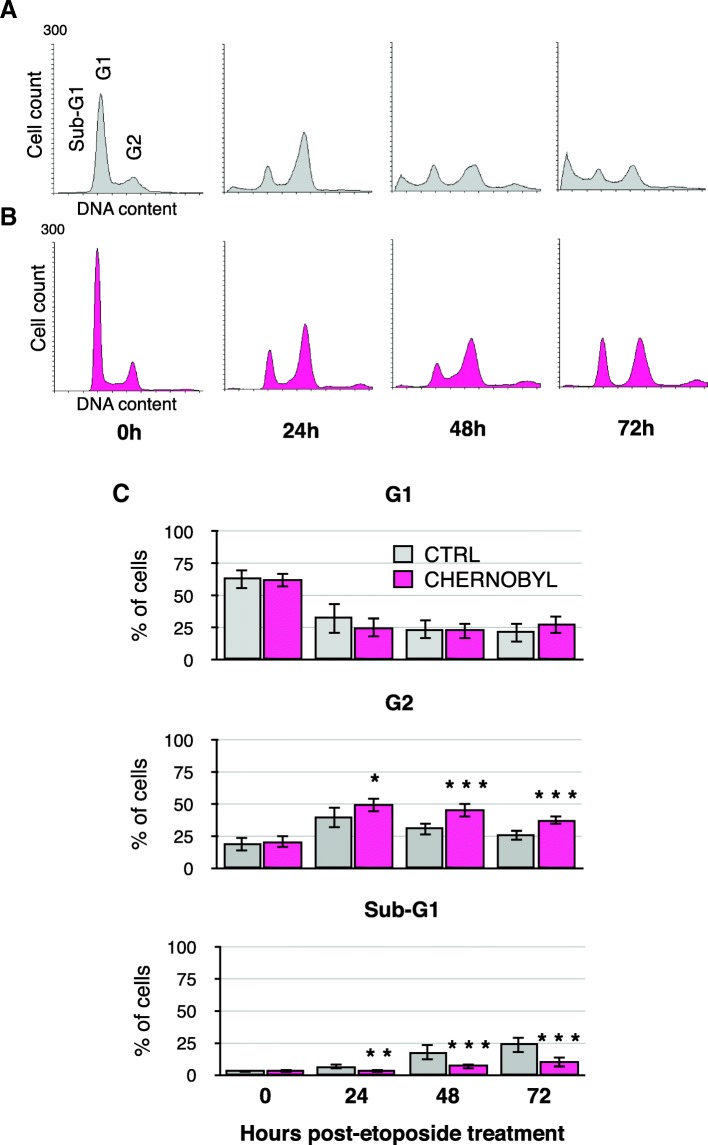
After DNA damage Chernobyl fibroblasts arrest stably at G2 phase. Example propidium iodide flow cytometry profile for one **a**.) control and **b**. Chernobyl cell line after 24, 48, and 72 h from etaposide treatment. **c**. The bar charts show the percentage of cells in G1, G2, and sub-G1 cell cycle phase for Chernobyl and control fibroblasts according to their DNA content by flow cytometry after 24, 48, and 72 h from etoposide treatment. The location of cells in sub-G1 (dying cells), G1, and G2 cell cycle phase are marked in the DNA histogram. The bar chart results are from three separate experiments using eight Chernobyl (*N* = 24) and eight control (*N* = 24) cell lines, variation is shown by standard deviation, and statistical analysis was done with Student’s t-test (* = *p* ≤ 0.05, ** = p ≤ 0.01, *** = *p* ≤ 0.001)

Chernobyl fibroblasts might withstand higher concentrations of DNA damaging drugs because they either had enhanced DNA repair capacity or were able to arrest longer to repair the damage. We tested the fibroblasts’ ability to repair DNA damage by host-cell reactivation assay on oxidized, nicked, and linearized plasmid substrates. Our results showed that there was no significant difference in repair efficiencies between Chernobyl and control fibroblasts (Additional file 2). Next, we tested the cell cycle effects of etoposide-mediated DNA damage on the fibroblasts. We treated cells with 20 μM of etoposide for 24 h and collected samples 24, 48, and 72 h from the start of the treatment for propidium iodide flow cytometry. Most of the cells arrested at the G2 phase (with 2 N DNA content) within 24 h after treatment. The control cells started to die (sub-G1) visibly more 2 days after the treatment (Fig. 4a and b). Etoposide is known to cause apoptosis [18] and according to Annexin V staining this was also observed in bank vole fibroblasts (Additional file 3). On average, Chernobyl fibroblasts were able to stay arrested at G2 longer than the control cell lines and thus avoiding cell death (Fig. 4c). Significantly more control cells were dying already 24 h after the treatment (x_CTRL_ = 6.5%, x_CHERNOBYL_ = 3.0%, Student’s t-test *p*-value = 0.001) and after 2 days there was, on average, about three times as many control cells dying as Chernobyl cells (x_CTRL_ = 17.5%, x_CHERNOBYL_ = 6.9%, *p*-value = 0.00008). There were no significant differences between the cell lines in amount of cells remaining at G1 (i.e., with 1 N DNA content) at any time point. The control cells’ inability to remain at G2 phase together with the observation of their increased cell death suggests that Chernobyl fibroblasts may have an intact DNA damage signaling pathway that leads to cell cycle arrest and lower sensitivity to apoptosis.

### After DNA damage p53-target transcripts are induced more in control than in Chernobyl fibroblasts

**Fig. 5:**
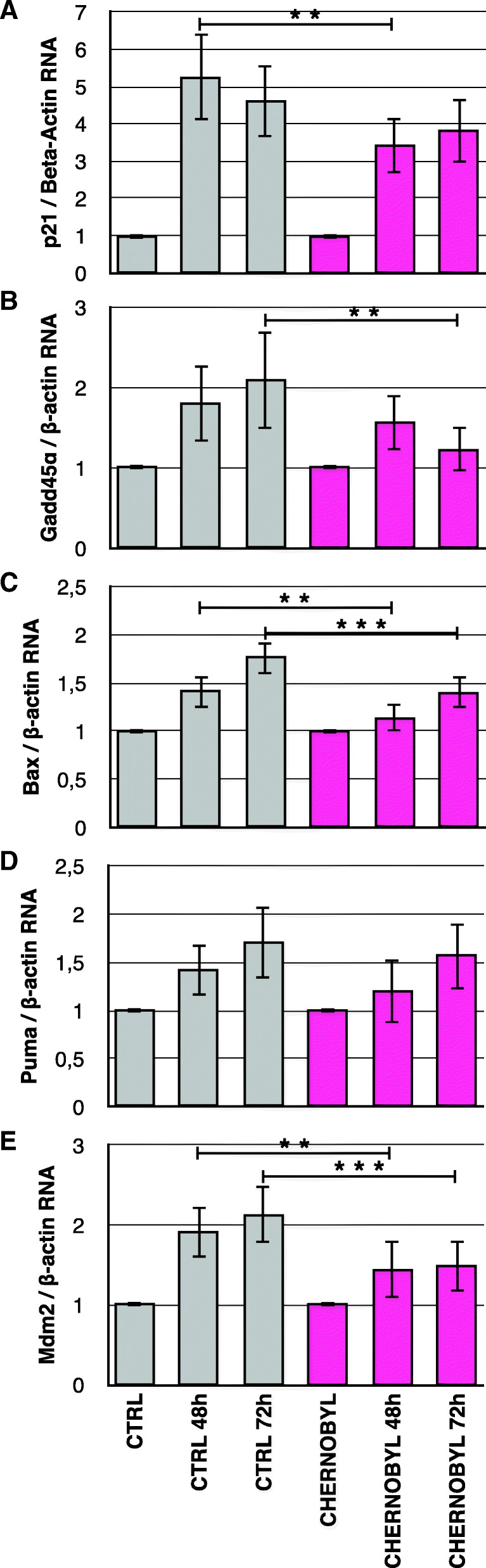
P53-target genes are induced more strongly in control cells than in Chernobyl bank vole fibroblasts. RNA was collected from untreated and etoposide-treated (20 μM) cells 48 and 72 h after the drug treatment. CDNA was prepared and quantitative-PCR was run with gene specific primers, and transcript levels were standardized to beta-actin transcript. Results are shown as a fold increase from untreated samples for **a**.) p21, **b**.) Gadd45α, **c**.) Bax, **d**.) Puma, and **e**.) Mdm2. The results are from three separate experiments using the eight Chernobyl (*N* = 24) and eight control cell lines (*N* = 24), variation is shown by standard deviation, and statistical analysis was done with Student’s t-test (** = *p* ≤ 0.01, *** = *p* ≤ 0.001)

Activation of transcription factor p53 by DNA damage can lead to apoptosis, senescence or cell cycle arrest. We collected etoposide-treated (20 μM of etoposide for 24 h) samples 48 and 72 h post-treatment for cDNA preparation. We quantified the transcript levels of p53-target genes: cell cycle regulators p21 and Gadd45α, pro-apoptotic proteins Puma and Bax, and p53-inhibitor Mdm2 and standardized them to beta-actin transcripts. Results are shown as fold change from untreated sample (Fig. 5). Induction of Bax and Mdm2 was significantly higher at both time points in control fibroblasts in comparison to Chernobyl cells (*p*-value_BAX-48h_ = 0.0021, *p*-value_BAX-72h_ = 0.00036, *p*-value_MDM2-48h_ = 0.0099, *p*-value_MDM2-72h_ = 0.00096; Fig. 5c and e). P21 was significantly more induced in control than Chernobyl cells at 48 h (*p*-value = 0.028; Fig. 5a) and Gadd45α at 72 h time point (*p*-value = 0.0016, Fig. 5b). No significant differences in Puma induction levels were detected at either time point (Fig. 5d). The amount of pro-apoptotic Bax transcript induction was about 20% higher in control than in Chernobyl cell lines, which is consistent with the higher percentage of dying control cells seen in the flow cytometry experiment.

## Discussion

Fibroblasts from Chernobyl bank voles were, on average, more resistant to radiation, oxidant treatment and DNA damaging drugs than the control fibroblasts. The DNA damaging drugs we used, etoposide, doxorubicin, and cisplatin have all been suggested to cause oxidant byproducts [19–21], thus the higher antioxidant levels of the Chernobyl fibroblasts’ may have also protected them against the indirect effects of these DNA damaging drugs. Neither control nor Chernobyl cell lines were homogenous in their responses to any of the experimental treatments; there were always one or two moderately resistant control and sensitive Chernobyl cell line, as is evident from the large variance of the experiments. This variability may indicate that the resistant traits are naturally occurring genetic variations. Bank vole populations at Chernobyl are from many different source populations and thus do host much genetic variation that may provide useful adaptive traits [12, 22].

Our results suggest that the environmental radiation in Chernobyl may select for adaptive cellular qualities in bank voles that defend against oxidants and help recover from DNA damage. However, statistical support for population level adaptation would require larger sample sizes from several different bank vole populations. Nevertheless, no-one has yet presented changed nucleotide substitution rates in the nuclear genomes of any Chernobyl inhabiting organisms. Mutations, as manifested by chromosomal abnormalities and phenotypic changes, have been reported in diverse Chernobyl taxa [2]. Although Baker et al. [17] have shown increased mutation rate of mitochondrial genomes in bank voles inhabiting Chernobyl, the results are not directly applicable to the nuclear genome because of differences in the DNA repair mechanisms and oxidative processes and conditions between nucleus and mitochondria. We found that Chernobyl fibroblasts withstood especially well chronic doses of dsDNA break inducing drugs (doxorubicin and etoposide) and, unlike control fibroblasts, they were able to stably arrest after DNA damage at G2 phase, when homologous recombination, the most accurate mechanism to repair dsDNA breaks, is most active. Thus, the implication of our results is the potential for bank vole cells to mount an efficient cellular mechanism against DNA breaks produced by radiation.

Chernobyl fibroblasts had more antioxidants in standard cell culture conditions than control cells and they were able to grow in higher oxidant concentrations. Chernobyl bank voles’ darker fur was hypothesized by Boratyński et al. [23] to be indicative of preferential production of eumelanin instead of pheomelanin, of which biosynthesis reduces reserves of antioxidant glutathione. Galván et al. [24] showed that Chernobyl birds living in areas of high radiation have increased glutathione levels, which correlated positively with improved body condition and reduced DNA damage. The antioxidant levels in Chernobyl birds have also been reported to be depleted, possibly due to the chronic radiation exposure leading to increased amounts of oxygen radicals [25, 26]. The unchallenged antioxidant levels were not tested in these ecological studies, but the results do suggest that antioxidants are one of the key factors in response against environmental radiation. Cells raise antioxidant levels following oxidant exposure and this reaction has been shown to be a crucial cellular response against low-dose radiation [27, 28]. Consequently, priming cells with oxidant or irradiation that increases antioxidant levels has shown to protect cells from a second exposure to radiation [27, 29]. Our results show that Chernobyl bank vole fibroblasts seem to be primed against abnormally high levels of oxidants.

P53 is a transcription factor and its activity (or lack of it) after genotoxic stress often determines whether the cell will arrest, commit apoptosis, or proceed into senescence (or mitotic catastrophe). P53 is activated by phosphorylation and the length and level of the activation together with other regulatory factors and cellular condition will decide cell fate. Low-dose chronic radiation has been shown to activate p53 and induce premature senescence in primary endothelial cells and fibroblasts [28, 30]. After p53 activating etoposide treatment Chernobyl cells were able to arrest stably at G2 whereas control cells started to die. Our results suggest that the higher induction levels of pro-apoptotic Bax were sufficient to induce apoptosis in control cell lines yet the lower levels of cell cycle regulators, p21 and Gadd45α, were high enough for the Chernobyl fibroblasts to remain at G2. Similar changes in amounts of p53-target gene transcripts have had a major impact on cell death in cancer cell lines with mutated p53 [31, 32]. Furthermore, p53 has been shown to have a role also in population level adaptation of mole rats to hypoxic environments [33]. However, in 1999 DeWoody [34] examined a short segment of Chernobyl bank voles’ p53 gene and found no apparent genetic changes in the sequence.

## Conclusions

Compared to control fibroblasts, fibroblasts isolated from bank voles inhabiting Chernobyl nuclear power plant accident site have elevated basal antioxidant levels and show decrease susceptibility to apoptosis after DNA damaging treatment. Accordingly, the Chernobyl fibroblasts were more resistant against oxidative and chronic DNA stresses.

Environmental radiation research is complicated by numerous interactions between exposed animal and its exposed environment. Meta-analysis has shown that organisms inhabiting Chernobyl area are approximately eight times more sensitive to radiation than species exposed to similar levels of radiation in controlled laboratory experiments [35]. Thus, the harmful effects of radiation apparently escalate with increased biological interaction and complexity. These ecological interactions might explain why some bank voles inhabiting Chernobyl nuclear accident site have two cellular mechanisms, high antioxidant levels and insensitivity to apoptosis, to cope with seemingly minor excess of environmental radiation.

## Methods

### Cells

Cells were isolated from eight male bank voles from the Chernobyl exclusion zone (GPS coordinates N51.44536 E30.06522) and eight male voles from Kiev area (N50.525859 E30.436933) (Additional file 4). Bank voles were caught using Ugglan Special2 multiple-capture live traps (Grahnab, Sweden) during June of 2016. The level of soil radiation at the trapping sites were measured at one cm above the ground using a Geiger-Mueller dosimeter (Inspector, International Medcom INC). Selected bank voles were of similar size (length: x_CTRL_ = 24.5 cm, x_CHERNOBYL_ = 21.9 cm, Student’s t-test *p*-value = 0.06; weight: x_CTRL_ = 88.7 g, x_CHERNOBYL_ = 92.1 g Student’s t-test *p*-value = 0.08). Animals were euthanized by cervical dislocation and all the procedures were performed in accordance with international guidelines and regulations for the use of animals in research. The study was approved by the Finnish Ethical Committee (license number ESAVI/7256/04.10.07/2014).

Skin fibroblasts were isolated 1–4 h after the animals were sacrificed according to the protocol of Seluanov [36]. In short, about one cm^2^ area of shaved skin from armpit of the animal was clipped and put into PBS for 10 min and then cut into small pieces and incubated at 37 °C in DMEM (30%) / PBS (70%) / Liberase DL Research Grade Enzyme (500 μg/ml) (Sigma-Aldrich) for 30 min. The tissue was filtered through 70 μm nylon mesh and placed into cell culture tube with DMEM (high glucose, GlutaMAX™) / 20% FBS / penicillin (100 U/ml) / streptomycin (100 μg/ml) / amphotericin B (1 μg/ml) (all from Thermofisher) and incubated at 37 °C for 2 days.

Cells were routinely cultured at 36 °C / 80% humidity / 5% CO_2_ in DMEM / 10% FBS / penicillin / streptomycin / amphotericin B. All experiment were done with similar passage numbers for both control and Chernobyl cells between passages 5–30. After cell passage 30 some of the cell lines showed chromosomal number abnormalities.

### Cell treatments

For cell treatments, 20,000 cells were plated to 24 well plates and let grow for 1 day to about 80% confluency before the treatment. Tert-butyl hydroperoxide (Alfa Aesar) was diluted in PBS and added to cells at concentrations of 150, 125, 100, 75, and 50 μM for one dose experiments and at 50, 40, 30, 20, and 10 μM for long exposure. Cisplatin (Sigma-Aldrich) was solubilized into 0.90% NaCl and added to cells at concentrations of 50, 40, 30, 20, and 10 μM for one dose experiments and at 25, 20, 15, 10, and 5 μM for long exposure. Doxorubicin (Sigma-Aldrich) was solubilized into DMSO and added to the cells at concentration of 160, 80, 40, 20, 10 μM for one dose experiments and at 20, 15, 10, 5, 1 μM for long exposure. Etoposide (Cayman Chemical) was solubilized into DMSO and added to cells at concentrations of 200, 100, 50, 25, and 12.5 μM for one dose experiments and at 45, 35, 25, 15, and 5 μM for long exposure. For one dose experiments the cells were incubated in the drug for 24 h after which fresh media was changed and cells were let to recover for 7 days before scoring the 100% confluent wells. For longer exposures, cells were treated with the drug every other day for four times by removing the old media and adding a new drug dose in fresh media. The 100% confluent wells were scored a day after the last treatment. Experimental controls were treated only with the drug solubilization solution.

For irradiation 20,000 cells were plated to cell culture tubes (NuncTM) and irradiated the next day with 10 Gy at the rate of 3.5 Gy/min from Cesium-137 source (Gammacell 2000). The cells were allowed to recover from the irradiation for 24 h and then replated to 25 cm^2^ plates. Cells were allowed to grow until they were 100% confluent.

### Total antioxidant assay

For each cell line, 40,000 cells were plated on 6 well plates and allowed to grow for 2 days before adding tert-butyl hydroperoxide for final concentration of 25 μM (or the same volume of PBS to experimental controls). Total antioxidant levels were measured 24 h after the treatment with OxiSelectTM Total Antioxidant Capacity Assay kit (Cell Biolabs) and the protein concentration was measured with Bio-Rad Protein Assay.

### Host-cell reactivation assay

Five thousand cells were plated on 96 well plate, treated next day with 20 μM etoposide for 8 hours, and then transfected with pGL3 (Promega) plasmid treated either with Nb.BsmI that nicked the plasmid coding sequence three times, with HindIII that linearized the plasmid after promoter sequence, or with 50 μM FeSO4 and 1 mM H2O2, which created oxidative damage on the plasmid. To control transfection efficiency cells were transfected also with pNL1.1 nano-luc vector. Luciferase expression was analysed 24 h after transfection with Nano-Glo Dual-Luciferase reporter assay system as suggested by the manufacturer (Promega).

### Quantitative-PCR

Total RNA was isolated with TRIzol® Reagent (Thermofisher) as suggested by the manufacturer. CDNA was prepared from 0.5 μg of RNA using RevertAid H Minus First Strand cDNA Synthesis Kit (Thermofisher) with random primers as suggested by the manufacturer. Quantitative-PCR was done with 5xHOT FIREPOL® Evagreen® qPCR Mix Plus (SolisBiodyne) as suggested by the manufacturer. Primers were designed to span at least one intron and were as follows; beta-actin: forward 5’ TGCGTGACATCAAAGAGAAG and reverse 5’GATGCCAGAAGATTCCATA, p21: forward 5’CTCCTGTGGGCACTTTAGGG and reverse 5’TGTCGCTGTCCTGCACTCT, Gadd45α: forward 5’CTGGAGGAAGTGCTCAGCAA and reverse 5’GGTCGACATTGAGCAGCTTG, Bax: forward 5’TGCCCGAGTTAATCAGAACCA and reverse 5’GGACTCCAGCCACAAAGATAGT, Puma: forward 5’CGGAGACAAGAAGAGCAGCA and reverse 5’TAGTTGGGCTCCATTTCGGG, Mdm2: forward 5’CAGTCTGAGTGAAGATGGGCA and reverse 5’AGCTAAGGAGATCTCAGGGTCT, p53: forward 5’CCAACACAAGCTCCTCTCCC and reverse 5’ATTCGCGTCCTGAGCATCC (metabion international AG and Eurofins Genomics). For each gene the PCR product was cloned into pJet-plasmid as recommended by CloneJET PCR Cloning kit manual and used in qPCR to make a standard curve. Results were analyzed with LightCycler® 96 system software.

### Propidium iodide flow cytometry

Forty thousand cells were plated on 6 well plates and treated with 20 μM etoposide the next day. 24 h after the treatment fresh cell culture media was changed and first samples were collected to 70% EtOH. Similarly 48 h and 72 h post-treatment samples were collected and stored in ethanol. Before the flow cytometry cells were resuspended into propidium iodide (100 μg/ml) and RNAse A (100 μg/ml) (Thermofisher). For each sample 10,000 cells were recorded with FACSCalibur. Flow cytometry results were analyzed with Flowing Software 2.5.1.

## Additional files

Additional file 1: Chernobyl and control fibroblasts are able to adjust to constant exposure to small concentrations of oxidant. The oxidant was added every other day for four times before scoring the wells that were 100% confluent a day after the last exposure. The results are from three separate experiments using the eight Chernobyl (*N* = 24) and eight control cell lines (*N* = 24). Variation is shown by standard deviation. (PDF 28 kb)

Additional file 2: The repair efficiency of nicked, oxidized, or linear plasmids is similar in control and Chernobyl bank vole fibroblasts. For host-cell reactivation assay, 5000 cells were plated on 96 well plate, treated next day with 20 μM etoposide for 8 hours, and then transfected with pGL3 (Promega) plasmid treated either with Nb.BsmI that nicked the plasmid coding sequence three times, with HindIII that linearized the plasmid after promoter sequence, or with 50 μM FeSO4 and 1 mM H2O2, which created oxidative damage on the plasmid. To control transfection efficiency cells were transfected also with pNL1.1 nano-luc vector. Luciferase expression was analysed 24 h after transfection with Nano-Glo Dual-Luciferase reporter assay system as suggested by the manufacturer (Promega). The bar charts show the relation of standardized treated to standardized untreated plasmid expression. The results are from four separate experiments using the eight Chernobyl (*N* = 32) and eight control cell lines (*N* = 32). Variation is shown by standard deviation. (PDF 27 kb)

Additional file 3: Etoposide induces apoptosis in bank vole fibroblasts. We treated the cells with DMSO or 20 μM of etoposide for 24 h, replaced the media, and collected samples 72 h post-treatment for propidium iodide and Annexin V flow cytometry with eBioscience Annexin V apoptosis Detection kit FITC as recommended by the manufacturer. The figure shows one control and one Chernobyl cell line. The percentage of healthy cells are shown in the lower-left corner, necrotic cells in the upper-left corner, and apoptotic cells at right. (PDF 66 kb)

Additional file 4: The trapping locations of the bank voles used in this study for fibroblast isolation. The green circles present the location at Kiev control area (average site radiation 0.2 μSv/h) where the control voles were trapped and the red circle denotes the site where Chernobyl voles were caught (average site radiation 21 μSv/h). Black dashed line indicates the 30 km Chernobyl exclusion zone. CNPP with a red triangle shows the location of the Chernobyl nuclear power plants. A map of Ukraine as an inset show by a red square the location of Chernobyl area. Map was created with ESRI ArcGIS 10.0. Satellite imagery © CNES/Airbus DS, Earthstar Geographics. Source: Esri, DigitalGlobe, GeoEye, i-cubed, Earthstar Geographics, CNES/Airbus DS, USDA, USGS, AEX, Getmapping, Aerogrid, IGN, IGP, swisstopo, and the GIS User Community | Esri, HERE, DeLorme. (PDF 106 kb)
