# Supplementary figures and images for: Fibroblasts from bank voles inhabiting Chernobyl have increased resistance against oxidative and DNA stresses
Source: BMC Cell Biol. 2018 Aug 29;19:17. doi: 10.1186/s12860-018-0169-9 (PMC6114495; doi:10.1186/s12860-018-0169-9)

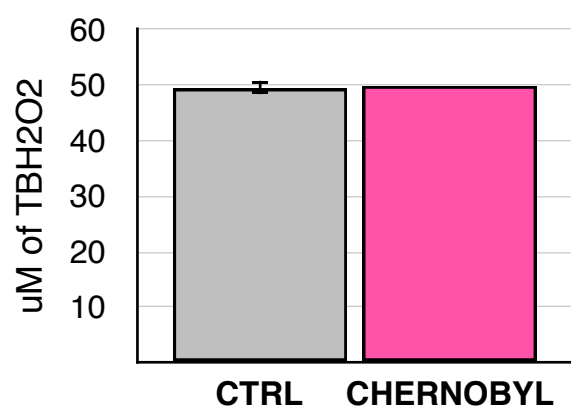

Supplement: Supplementary file 1 — Chernobyl and control fibroblasts are able to adjust to constant exposure to small concentrations of oxidant. The oxidant was added every other day for four times before scoring the wells that were 100% confluent a day after the last exposure. The results are from three separate experiments using the eight Chernobyl (N = 24) and eight control cell lines (N = 24). Variation is shown by standard deviation. (PDF 28 kb) [file 12860_2018_169_MOESM1_ESM.pdf]

**A.**

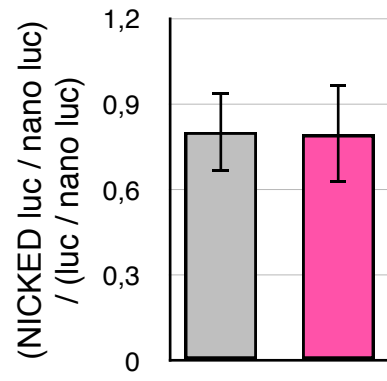

**B.**

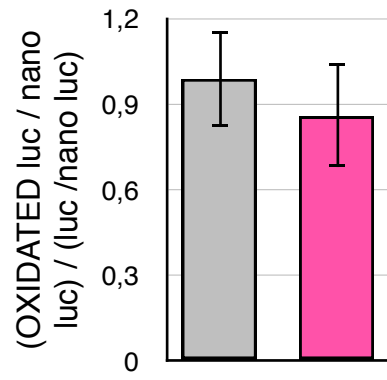

**C.**

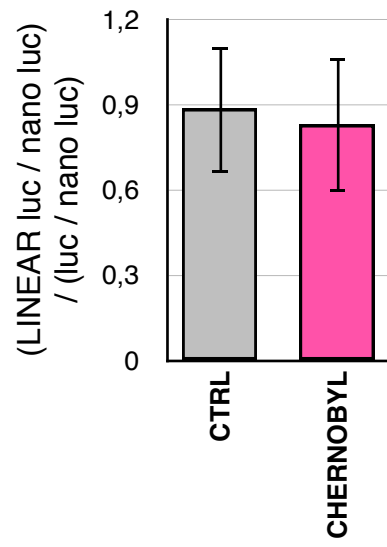

Supplement: Supplementary file 2 — The repair efficiency of nicked, oxidized, or linear plasmids is similar in control and Chernobyl bank vole fibroblasts. For host-cell reactivation assay, 5000 cells were plated on 96 well plate, treated next day with 20 μM etoposide for 8 hours, and then transfected with pGL3 (Promega) plasmid treated either with Nb.BsmI that nicked the plasmid coding sequence three times, with HindIII that linearized the plasmid after promoter sequence, or with 50 μM FeSO4 and 1 mM H2O2, which created oxidative damage on the plasmid. To control transfection efficiency cells were transfected also with pNL1.1 nano-luc vector. Luciferase expression was analysed 24 h after transfection with Nano-Glo Dual-Luciferase reporter assay system as suggested by the manufacturer (Promega). The bar charts show the relation of standardized treated to standardized untreated plasmid expression. The results are from four separate experiments using the eight Chernobyl (N = 32) and eight control cell lines (N = 32). Variation is shown by standard deviation. (PDF 27 kb) [file 12860_2018_169_MOESM2_ESM.pdf]

**CTRL**

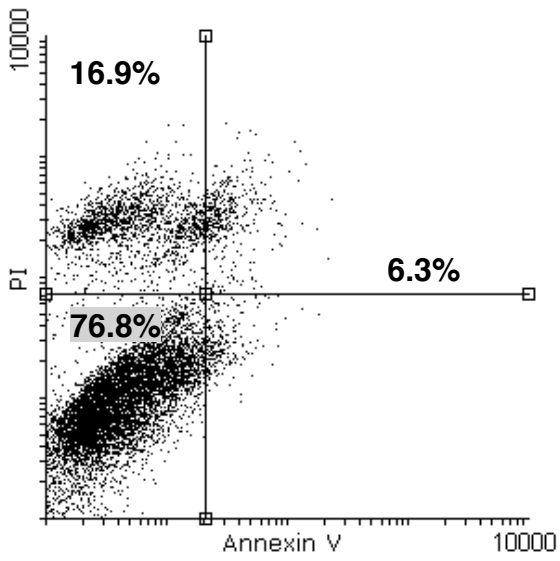

**CTRL + 20 $\mu$ M Etoposide**

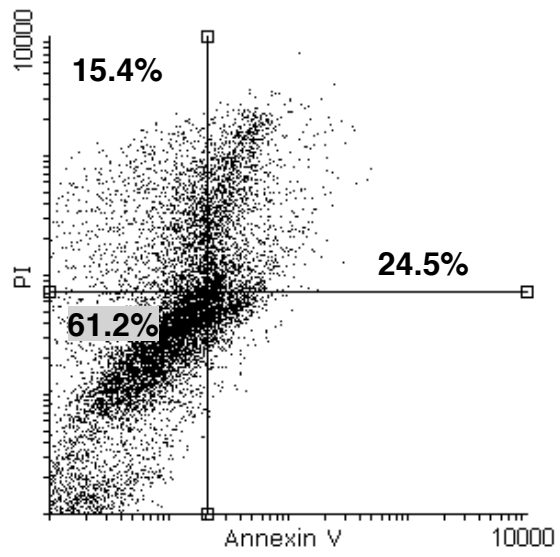

**CHERNOBYL**

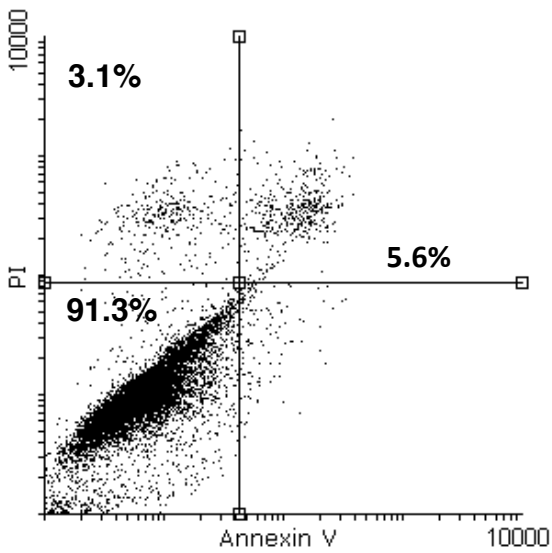

**CHERNOBYL + 20 $\mu$ M Etoposide**

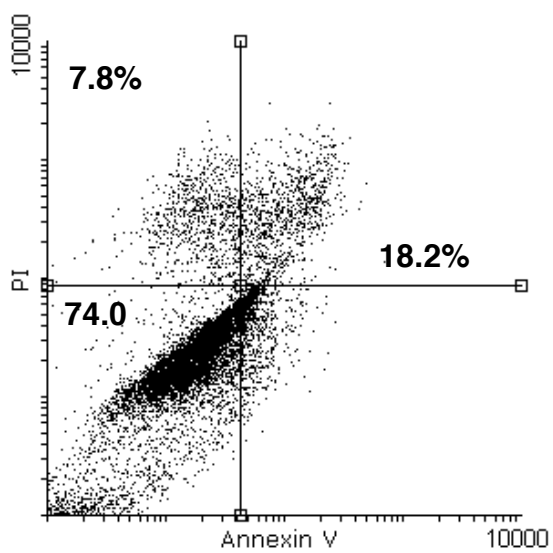

Supplement: Supplementary file 3 — Etoposide induces apoptosis in bank vole fibroblasts. We treated the cells with DMSO or 20 μM of etoposide for 24 h, replaced the media, and collected samples 72 h post-treatment for propidium iodide and Annexin V flow cytometry with eBioscience Annexin V apoptosis Detection kit FITC as recommended by the manufacturer. The figure shows one control and one Chernobyl cell line. The percentage of healthy cells are shown in the lower-left corner, necrotic cells in the upper-left corner, and apoptotic cells at right. (PDF 66 kb) [file 12860_2018_169_MOESM3_ESM.pdf]

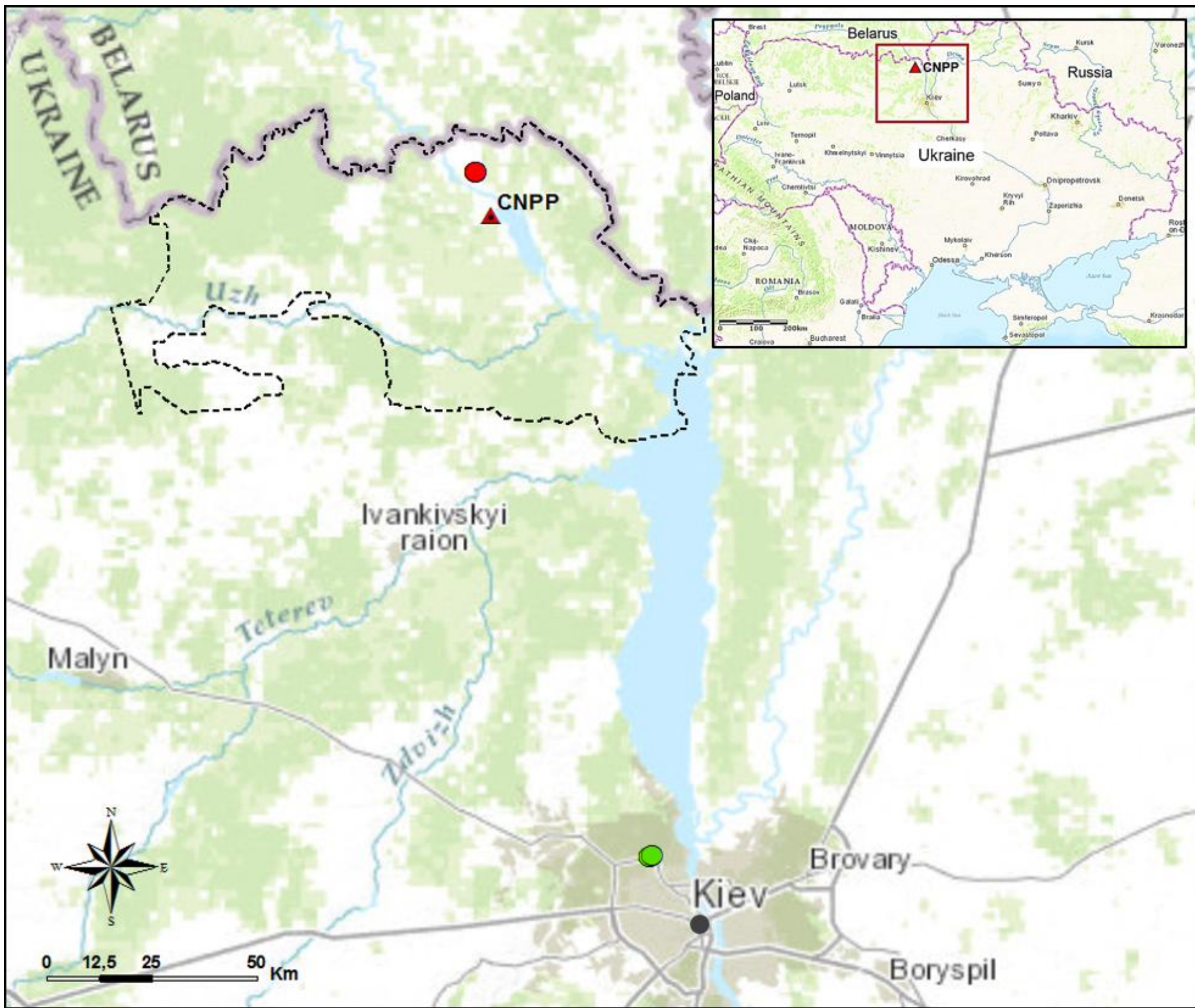

Supplement: Supplementary file 4 — The trapping locations of the bank voles used in this study for fibroblast isolation. The green circles present the location at Kiev control area (average site radiation 0.2 μSv/h) where the control voles were trapped and the red circle denotes the site where Chernobyl voles were caught (average site radiation 21 μSv/h). Black dashed line indicates the 30 km Chernobyl exclusion zone. CNPP with a red triangle shows the location of the Chernobyl nuclear power plants. A map of Ukraine as an inset show by a red square the location of Chernobyl area. Map was created with ESRI ArcGIS 10.0. Satellite imagery © CNES/Airbus DS, Earthstar Geographics. Source: Esri, DigitalGlobe, GeoEye, i-cubed, Earthstar Geographics, CNES/Airbus DS, USDA, USGS, AEX, Getmapping, Aerogrid, IGN, IGP, swisstopo, and the GIS User Community | Esri, HERE, DeLorme. (PDF 106 kb) [file 12860_2018_169_MOESM4_ESM.pdf]
